# An Improved Algorithm of Congruent Matching Cells (CMC) Method for Firearm Evidence Identifications

**DOI:** 10.6028/jres.120.008

**Published:** 2015-04-29

**Authors:** Mingsi Tong, John Song, Wei Chu

**Affiliations:** 1 School of Mechatronics Engineering, Harbin Institute of Technology, Harbin 150001, China; 2 National Institute of Standards and Technology, Gaithersburg, MD 20899 USA

**Keywords:** ballistics identification, cartridge case, Congruent Matching Cells (CMC), correlation cells, forensic science, image processing

## Abstract

The Congruent Matching Cells (CMC) method was invented at the National Institute of Standards and Technology (NIST) for firearm evidence identifications. The CMC method divides the measured image of a surface area, such as a breech face impression from a fired cartridge case, into small correlation cells and uses four identification parameters to identify correlated cell pairs originating from the same firearm. The CMC method was validated by identification tests using both 3D topography images and optical images captured from breech face impressions of 40 cartridge cases fired from a pistol with 10 consecutively manufactured slides. In this paper, we discuss the processing of the cell correlations and propose an improved algorithm of the CMC method which takes advantage of the cell correlations at a common initial phase angle and combines the forward and backward correlations to improve the identification capability. The improved algorithm is tested by 780 pairwise correlations using the same optical images and 3D topography images as the initial validation.

## 1. Background

In forensic investigations involving firearms, toolmarks on bullets and cartridge cases fired from a firearm are important clues and evidence. Comparisons of these toolmarks have more than a hundred years of history through the use of manual comparison microscopes [1]. In recent years, automated identification technologies and systems have been developed [2, 3]. For correlations of the cartridge case, most identification systems correlate the whole region without taking into account the fact that not all regions on the primer are well-marked by the impression of the breech face of the gun slide during firing. Some regions of the primer that make poor contact with the breech face may generate “invalid” areas of poor correlation which may affect the capability of identification. In 2012, the Congruent Matching Cells (CMC) method was invented at the National Institute of Standards and Technology (NIST) to provide objective and high-accuracy ballistics identification and evidence searches [4]. The CMC method divides the measured image into small correlation cells so that “invalid” areas can be isolated from “valid” areas that contain unique identifying marks in order to improve correlation accuracy. The method uses four identification parameters to determine the number of valid congruent matching cell pairs (CMC) contained in a pair of images. A numerical identification criterion, *C* = 6, was suggested as a criterion for identifying images of matching cartridge cases which are fired from the same firearm [4]. Pairs of images with CMC greater than 6 are identified as matches, and pairs of images with CMC less than 6 are concluded to be non-matches.

The CMC method was initially tested using both three-dimensional (3D) topography images and optical grayscale intensity images of primers from a set of 40 test fires from 10 consecutively manufactured pistol slides. These measurements allowed for 780 pairwise correlations for both the 3D topography images and optical images. The test results showed that all image pairs are correctly identified without any false identification (false positive) or false exclusion (false negative) [5, 6]. However, the separation between the known-matching (KM) and known-non-matching (KNM) distributions of the number of CMCs for the optical images pair is not significant [6] (see also Fig. 8a). In order to increase the correlation accuracy, this paper examines the processing of the CMC method. An improved algorithm is proposed that takes advantage of the cell correlations at a common initial phase angle and includes a forward and backward correlations strategy (i.e. A vs. B and B vs. A) to significantly improve the capability of identification. The improved algorithm is tested using both 3D topography images and optical images captured from breech face impressions from the same set of 40 cartridge cases.

## 2. Congruent Matching Cells (CMC) Method

### 2.1. Basic Concepts

Depending on the contact conditions with the gun parts, the surfaces of the bullets and cartridge cases fired from a firearm include both “valid” and “invalid” correlation regions [4]. A valid correlation region contains “individual characteristics” [7] of the ballistics signature that can be used effectively for identification. An invalid correlation region does not contain individual characteristics of the firearm’s ballistics signature and should be eliminated from identification. If the correlation is performed on the whole region of two correlated images, a quantitative measure of correlation may be relatively low, because large invalid correlation areas may be involved in the correlation. If instead, the correlation region is divided into cells for correlation, the valid correlation cells can be identified and the invalid correlation cells can be eliminated from correlations. This procedure can enhance the quantitative measure of correlation [4].

When images A and B originating from a pair of cartridge cases fired from the same firearm are compared, the cell pairs located in their common valid correlation area can be characterized by four identification parameters:

1. High correlation values quantified by the cross correlation function maximum, *CCF*_max_ [8];
2. Similar registration angles *θ*;
3. Similar *x*-*y* spatial distribution.

Four corresponding “thresholds” are used to identify the Congruent Matching Cells: *T*_CCF_, *T*_θ_, *T*_x_, and *T*_y_. If the registered cell pairs come from valid correlation areas of A and B originating from the same firearm, their correlation value *CCF*_max_ should exceed *T*_CCF_, and their deviations in rotation angle *θ* and lateral translation *x* and *y* at the matched position should all fall within ranges given respectively by *T*_θ_, *T*_x_, and *T*_y_, then those cell pairs are regarded as a congruent, matching set of cell pairs. Inspired by the numerical identification criterion of the Consecutively Matching Striae (CMS) method proposed by Biasotti and Murdock for identification of bullet striation signatures [9], the numerical identification criterion for the CMC method is suggested as *C* = 6 [4], i.e. When the CMC number is equal to or greater than 6, images A and B are concluded to be a match.

### 2.2. Original Algorithm of CMC Method

**Fig. 1:**
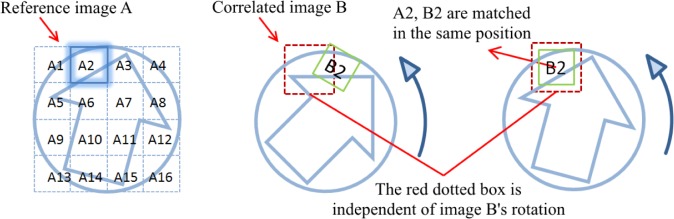
Correlation scheme using the fast CMC method: A_2_ and B_2_ represent the matched cell pair. During the correlation process, cell A_2_ only scans the red dotted window in each rotated position of the correlated image B [6].

In the original algorithm of the CMC method, the images are divided into a cell array. For a pair of compared images A and B, after eliminating void cells that contain zero or very few data points, each cell in the reference image A scans the correlated image B at each of its rotational positions. Then the *θ*, *x*, and *y* parameters of each cell are registered at the position of the maximum correlation value, *CCF*_max_ (see Fig. 1 which shows only a simplified 4 × 4 cells array), and are compared with the thresholds of *T*_CCF_, *T*_θ_, *T*_x_, and *T*_y_ to determine whether or not they are Congruent Matching Cells (CMC) [7]. To reduce the computation time, the median values of *θ*, *x*, and *y* are selected as virtual references for identification of the correlated cell pairs [5].

## 3. Improved Algorithm of CMC Method

As stated before, if two compared images originate from the same firearm, the cell pairs located in their common valid correlation area must have similar registration angles *θ*, which are close to the initial phase angle *Θ_0_*, where the two correlated images can be correctly matched [4]. In the beginning of the correlation, however, the initial phase angle is uncertain as the correlation is not performed and the orientation of cartridge case setup is unknown when images are captured. As a result, in the previous optical image validation tests, each cell in the reference image A must scan the correlated image B at each of its rotated positions and be registered only at the *CCF*_max_ position [6] without taking account of the initial phase angle. Ideally, for the pairwise cell correlations of matching image pairs, the maximum correlation value *CCF*_max_ should always be located at the angular position around the initial phase angle *Θ_0_*; and the CCF values obtained at other rotation angles should be smaller and should be ignored [4]. However, after extensive case studies, instances can be observed where some cell pairs from matching images are registered at an angle other than *Θ_0_*, leading to an incorrect correlation conclusion. This is most common when the CCF distribution patterns of the matching and non-matching cell correlations show large overlapping regions [6].

As an example, Table 1 shows the correlation results represented by the values of *CCF*_max_, *θ* and *x*, *y* of a KM pair of optical images. The nominal cell number is 49 (a 7 × 7 array of cells); after eliminating void cells, the effective cell number is *N* = 30; the rotation range is ±30° with 3° increments. The missing indexes in Table 1 and 2 represent the void cells [6]. Each row in the tables represents a pairwise cell correlation. Each row marked in red represents a CMC. For the 12 CMCs shown in Table 1, their registration angles *θ* are distributed from 12° to 18° with the thresholds set as *T*_CCF_ = 25 %, *T*_θ_ = ±3° and *T*_x_ = *T*_y_ = ±25 pixel with 5.06 μm pixel spacing [6]. The initial phase angle for this image pair taken as a whole is estimated to be *Θ_0_* = 15°. The other 18 pairwise cells are not congruent matching cells, since the rotation angle associated with their maximum CCF value did not fall within the threshold range of the registration phase angle from 12° to 18°.

However, due to the fact that CCF distribution patterns of matching and non-matching cell correlations show large overlapping regions [6], some of the valid cell pairs may be mistakenly excluded from the CMC count because by chance their correlation yields a higher CCF value at a rotation angle outside the threshold range *T*_θ_ = ±3° than the CCF value found within the range. In order to avoid the elimination of these valid cell pairs, we use the initial phase angle *Θ_0_* = 15° as a fixed registration angle. With the fixed registration angle, the CMC number will be determined only by three parameters (*CCF*_max_, *x*, and *y*). As the blue rows in Table 2 indicate, the CMC number increases from 12 to 15. It can be seen that the CCF values of the additional CMCs are just a little lower than the CCF values of the corresponding cells in Table 1. In addition, (two cells are considered as CMCs in Table 1, but not in Table 2. These cells will be included in the refined analysis discussed below. Based on these correlation results, a new algorithm using CMCs located at the initial phase angle, instead of the original algorithm using CMCs located in the full angular registration range, is proposed to improve correlation accuracy. The key question is how the initial phase angle *Θ_0_* is determined.

**Fig. 2:**
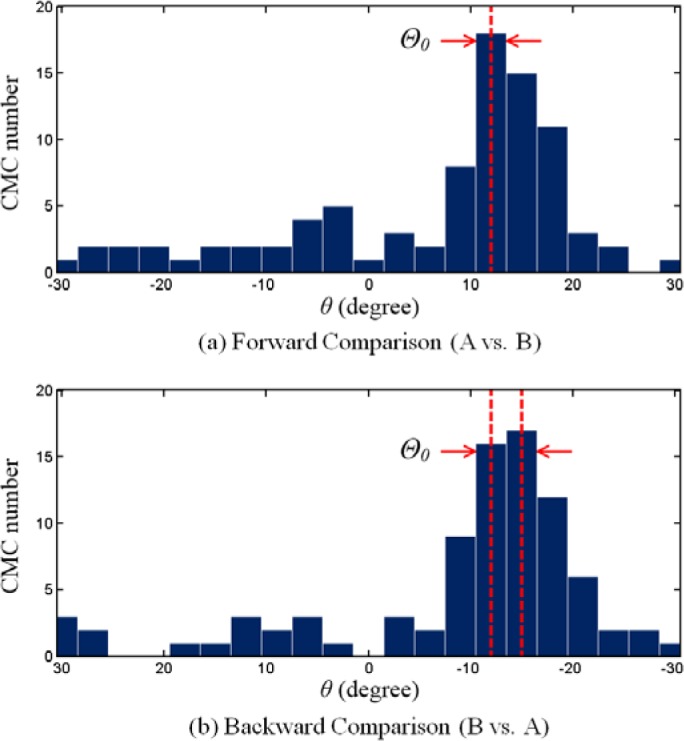
CMC-*θ* distribution of a typical correlation for a pair of KM optical images. Red lines indicate the angular range of “high CMCs”.

**Fig. 3:**
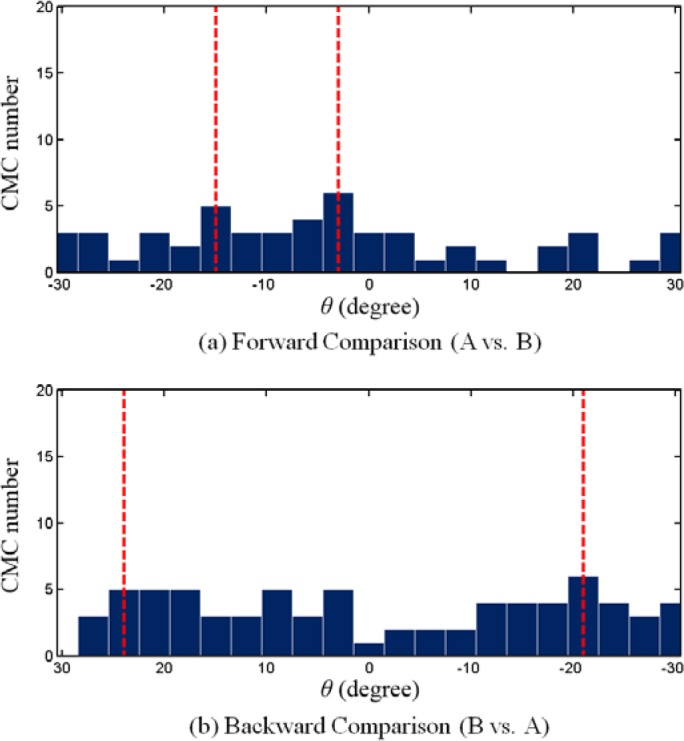
CMC-*θ* distribution of a typical correlation for a pair of KNM optical images. Red lines indicate the angular range of “high CMCs”.

Figure 2a shows the CMC-*θ* distribution for the above KM image pair at each registration angle *θ* between +30° and −30° with 3° increments. It shows a distribution which exhibits a single peak concentrated at 12°. If two images are truly matching, the CMC-*θ* distribution of matching image pairs should have a prominent peak located near the initial phase angle *Θ_0_*, while non-matching image pairs may have a relatively flat and random CMC-*θ* distribution pattern (Fig. 3a).

An additional criterion is now introduced to distinguish the matching from the non-matching distributions, such as those shown in Figs. 2 and 3. It can be seen that for the CMC-*θ* distribution of the KM correlation (Fig. 2a), the second highest CMC number is close to the maximum CMC number relative to its phase angle. While for the KNM correlation (Fig. 3a), the second highest CMC number could appear anywhere in the angle range. Thus, the first step for the improved algorithm is using an additional “high CMC number” along with the “maximum CMC number” to determine the angular position of the initial phase. If we define the “high CMC number” as (*CMC*_max_ − *τ*), where τ is an empirical constant (in this paper, we specify *τ* =1), and let the angular range of the “high CMCs” be the maximum angular interval with the corresponding CMC number is greater than or equal to the “high CMC number”, then the angular range of “high CMCs” in Fig. 2a is only the histogram bar centered around 12°. Therefore, the initial phase angle can be considered as 12°. However, for the KNM case as shown in Fig. 3a, the “high CMC number” is (*CMC*_max_ − 1 = 5), and the corresponding angular range of the “high CMCs” extends from −15° to −3°. The wide distribution range between the angles of the maximum and the high CMC number suggests that the KNM image pairs have no common initial phase angle for their angular registration.

**Fig. 4:**
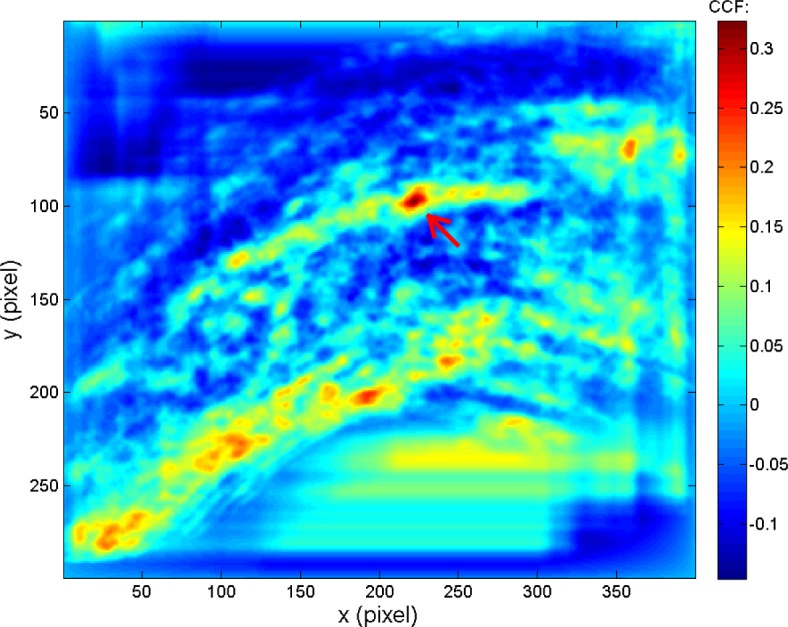
A typical CCF map on a known congruent matching cell of correlated KM optical image scanned by the corresponding correlation cell of the pairwise matched reference image.

**Fig. 5:**
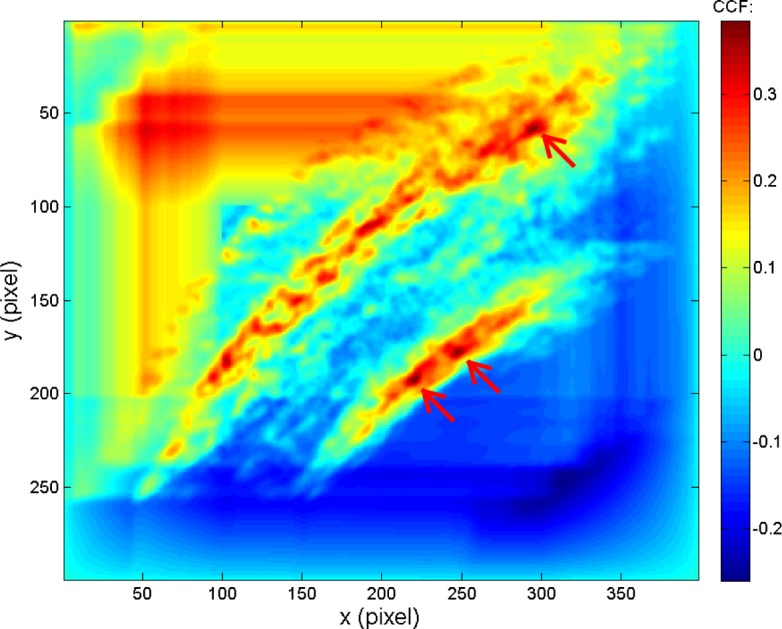
A typical CCF map on a cell of correlated KNM optical image scanned by a correlation cell of a non-matched reference image.

The CMCs in the KM image pairs result primarily from the individual characteristics which represent the similarity of their ballistics signatures. On the other hand, the CMCs in the KNM image pairs usually result from random factors, mainly because KNM surfaces are not expected to be correlated at all. For example, Fig. 4 shows a typical CCF map of a known congruent matching cell of a correlated KM image scanned by the corresponding correlation cell of the pairwise matched reference image. It can be seen that there is only one CCF peak above the threshold *T*_CCF_ = 25 %, indicated by the arrow. Figure 5 shows a typical CCF map for a KNM situation, a single cell of one image correlated with a cell of a non-matching reference image. It can be seen that there are at least three peaks with similar CCF values above the threshold. This example suggests that the CCF peak in the KM images is relatively systematic and stable. Its position and CCF value do not significantly change with the change of correlation conditions (cell size, cell incision angle, cell shift direction, etc.). Conversely, the CCF peaks in the KNM images are randomly oriented and are not stable. These KNM peak positions and CCF values may significantly change with correlation conditions, which may help distinguish the non-matching correlations.

**Fig. 6:**
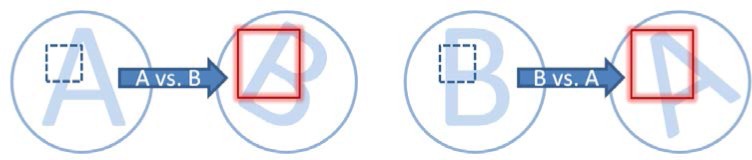
Depiction of the forward and backward correlations. For both directions a cell-size area in one image is scanned over a larger region of interest in the other image.

Based on the above observation that the KM distributions are less sensitive to correlation conditions than KNM distributions, the second proposed step is to combine the forward and backward correlations (i.e., A vs. B and B vs. A, see Fig. 6) and utilize the different features of CMC-*θ* distributions in KM and KNM images to detect and exclude random CMC peaks for non-matching correlations from the CMC-*θ* distributions. Figures 2b and 3b show the CMC-*θ* distributions of the backward correlations of the KM and KNM. The forward correlations are shown in Fig. 2a and Fig. 3a. It can be seen that the forward and backward CMC-*θ* distributions of the KM correlation are very similar. On the contrary, the CMC-*θ* distributions in the two KNM figures are significantly different because there is no common initial phase angle for the KNM image pairs. This suggests a method for improving the CMC algorithm by combining the angular ranges between the lines of high CMCs found for the forward and backward CMC-*θ* distributions. For example, in the KM correlation (Fig. 2), the angular range of the “high CMCs” for both the forward and backward distributions is from 12° to 15° which is close to that of the forward correlations 12°. However, for the KNM correlation in Fig. 3, the angular range of the “high CMCs” for the forward and backward distributions is expanded from (−15° to −3°, forward) to (−24° to 21°, backward). The wide distribution range suggests that there is not a common initial phase angle between these two KNM image pairs.

## 4. Experiment

The optical images and 3D topography images from the same set of 40 cartridge cases discussed in references [5, 6] are tested using the improved algorithm of the CMC method. Each set includes 63 KM and the 717 KNM image pairs for a total of 780 correlations. The first set includes 40 optical images captured using a microscope with a ring light; the second set of images includes 40 topography images captured using a confocal microscope.

Each set of images is processed using the following procedure:

1. Conduct both forward and backward correlations at each rotation and record the registration based on *CCF*_max_, *x*, and *y* for each cell at each rotation. These data will be used in the next two steps separately.
2. At every rotation angle, each cell in the reference image finds a registration position in the compared image with a maximum CCF value. By selecting the registration with the maximum CCF value for each cell, the two CMC numbers determined by the four thresholds can be obtained based on the original algorithm [6]. The lower CMC number is used as the initial result. An example of the results is shown in Table 1.
3. Build CMC-*θ* distributions using the data generated in step 1, by counting the number of cells that have congruent positions at each individual rotation angle. Calculate the angular range of “high CMCs” using both the forward and backward CMC-*θ* distributions, as illustrated in Figs. 2 and 3.
4. If the angular range of the “high CMCs” is within the range *T*_θ_, identify the CMCs for each rotation angle in this range and combine them to give the number of CMCs for this comparison in place of the original CMC number. In this step, if the range is narrower than *T*_θ_, the nearby angles are included to make the range equal to *T*_θ_; CMCs with same index in each rotation are only counted once.
5. If the angular range of the “High CMCs” is larger than the range *T*_θ_, keep the CMC value obtained from step 2 as the final CMC for this comparison.

**Fig. 7:**
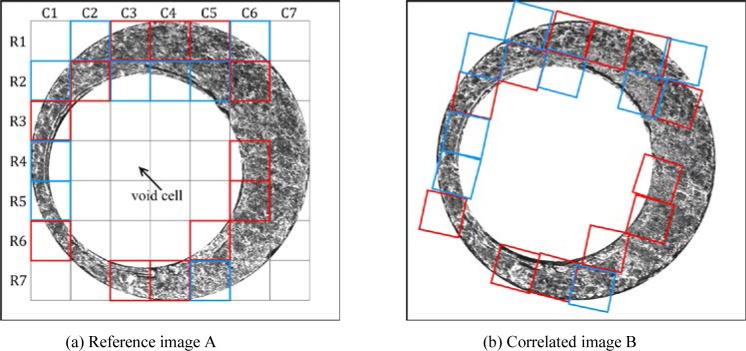
Shows the KM image pair. Red cells represent CMCs identified by the original algorithm; blue cells represent additional CMCs identified using the improved algorithm.

**Fig. 8:**
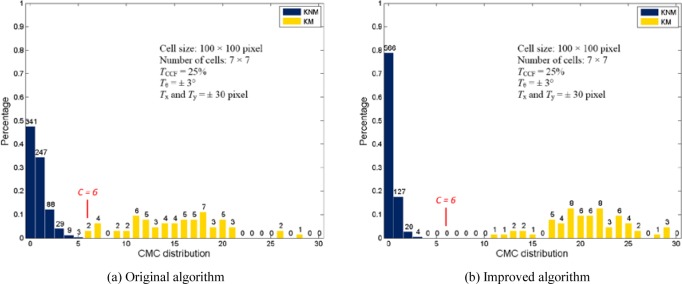
CMC distribution of the optical image set.

**Fig. 9:**
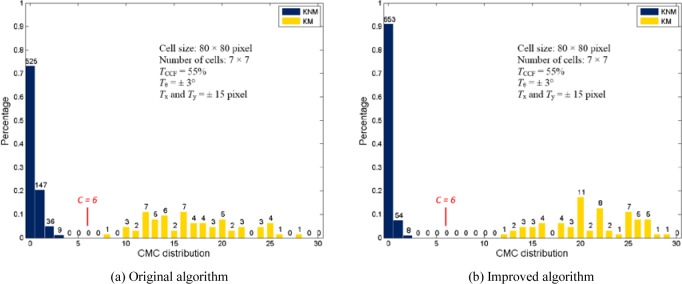
CMC distribution of the topography image set.

Table 3 and Fig. 7 show the final CMCs of the image pair used in section 3. Nine additional CMCs marked in blue are identified by the improved algorithm. Figures 8 and 9 show the results obtained from the original and improved algorithm. For the optical image set, the minimum CMC number of the KM correlations increases from 6 to 11. All of the 780 image correlations are identified correctly as matching or non-matching by the criterion *C* = 6 used in the improved algorithm. Similarly, for the topography image set, the minimum CMC number of the KM correlations increases from 8 to 12. In all of these results, the CMC values for KNM image correlations are consistently low in number. In summary, these experiments demonstrate that the improved algorithm can enhance the identification capability of the CMC method for both optical intensity and topographical breech face representations.

## 5. Discussion and Conclusion

The improved algorithm of the CMC method keeps account of the *CCF*_max_ values of cell correlation at each registration angle, rather than only the *CCF*_max_ value at a single registration angle, which may fall outside the accepted range of *T*_θ_. By using these additional values, the revised algorithm builds the CMC-*θ* distribution which represents the important features of the matching image pair and the non-matching image pair. For the matching image pair, correlation positions of cells are highly concentrated around a particular initial phase angle, mostly caused by the similarity of the individual characteristics of the KM image pairs. For the non-matching image pairs, the distribution of the correlation values seems random, irregular, and relatively flat. As the experimental results show, the improved algorithm of the CMC method can identify the matching cells with lower CCF values which may be otherwise ignored by the original algorithm, and therefore improves the capability of the CMC method to distinguish matching images from non-matching images.

In our future work, we will test different sample sets to test and optimize the stability and capability of the improved CMC method further.

## Figures and Tables

**Table 1:** A typical correlation for a pair of KM optical images (Angular registration from −30° to 30°).

Cell index (Col., Row)	*CCF_max_*	*θ* (degree)	[*x*, *y*] (pixel)
(C1, R2)	0.34707	−30	[3,−2]
(C1, R3)	0.29071	12	[18,−7]
(C1, R4)	0.30964	−12	[19,−24]
(C1, R5)	0.26825	−30	[30,15]
(C1, R6)	0.41703	12	[1,0]
(C2, R1)	0.38082	−3	[−7,6]
(C2, R2)	0.44403	15	[9,6]
(C2, R7)	0.44394	18	[−117,88]
(C3, R1)	0.33065	15	[6,1]
(C3, R2)	0.30452	−9	[−25,−27]
(C3, R7)	0.56988	15	[23,1]
(C4, R1)	0.38804	15	[4,0]
(C4, R2)	0.44964	24	[26,9]
(C4, R7)	0.35934	15	[24,−3]
(C5, R1)	0.36111	12	[19,1]
(C5, R2)	0.35324	12	[−98,−130]
(C5, R6)	0.50059	12	[8,4]
(C5, R7)	0.29452	−15	[−98,−130]
(C6, R1)	0.43035	9	[25,14]
(C6, R2)	0.44577	18	[−3,−14]
(C6, R3)	0.32158	0	[27,−124]
(C6, R4)	0.26124	18	[9,−19]
(C6, R5)	0.45302	15	[17,−6]
(C6, R6)	0.33633	18	[90,−166]
(C6, R7)	0.55679	−6	[119,84]
(C7, R2)	0.37795	−3	[−7,−13]
(C7, R3)	0.39892	−15	[−3,−3]
(C7, R4)	0.37137	−12	[−13,−144]
(C7, R5)	0.29614	21	[−127,181]
(C7, R6)	0.42114	−15	[−3,2]

**Table 2:** A typical correlation for a pair of KM optical images (Angular registration based on 15°).

Cell index (Col., Row)	*CCF_max_*	*θ* (degree)	[*x*, *y*] (pixel)
(C1, R2)	0.27635	15	[42,−65]
(C1, R3)	0.26147	15	[13,8]
(C1, R4)	0.25001	15	[−4,101]
(C1, R5)	0.26463	15	[19,11]
(C1, R6)	0.40851	15	[11,14]
(C2, R1)	0.29890	15	[−2,4]
(C2, R2)	0.44403	15	[9,6]
(C2, R7)	0.44390	15	[−119,81]
(C3, R1)	0.33065	15	[6,1]
(C3, R2)	0.28137	15	[10,−3]
(C3, R7)	0.56988	15	[23,1]
(C4, R1)	0.38804	15	[4,0]
(C4, R2)	0.36664	15	[13,−2]
(C4, R7)	0.35934	15	[24,−3]
(C5, R1)	0.29101	15	[−144,71]
(C5, R2)	0.33398	15	[17,−4]
(C5, R6)	0.45089	15	[19,−2]
(C5, R7)	0.25491	15	[100,−79]
(C6, R1)	0.38182	15	[53,38]
(C6, R2)	0.44428	15	[7,−4]
(C6, R3)	0.26104	15	[102,−123]
(C6, R4)	0.24524	15	[10,−8]
(C6, R5)	0.45302	15	[17,−6]
(C6, R6)	0.33438	15	[87,−154]
(C6, R7)	0.55393	15	[119,84]
(C7, R2)	0.31479	15	[−57,−81]
(C7, R3)	0.35473	15	[−168,−80]
(C7, R4)	0.31181	15	[7,−152]
(C7, R5)	0.24747	15	[−130,183]
(C7, R6)	0.41028	15	[52,134]

**Table 3:** Final CMCs in the KM correlation.

Cell index (Col., Row)	*CCF_max_*	*θ* (degree)	[*x*, *y*] (pixel)
(C1, R2)	0.29604	12	[20,−7]
(C1, R3)	0.29071	12	[18,−7]
(C1, R4)	0.27868	12	[16,−5]
(C1, R5)	0.26463	15	[19,11]
(C1, R6)	0.41703	12	[1,0]
(C2, R1)	0.29890	15	[−2,4]
(C2, R2)	0.44403	15	[9,6]
(C3, R1)	0.33065	15	[6,1]
(C3, R2)	0.28138	15	[10,−3]
(C3, R7)	0.56988	15	[23,1]
(C4, R1)	0.38804	15	[4,0]
(C4, R2)	0.36664	15	[13,−2]
(C4, R7)	0.35934	15	[24,−3]
(C5, R1)	0.36111	12	[19,1]
(C5, R2)	0.33398	15	[17,−4]
(C5, R6)	0.50059	12	[8,4]
(C5, R7)	0.27228	12	[6,5]
(C6, R1)	0.42877	12	[11,3]
(C6, R2)	0.44577	18	[−3,−14]
(C6, R4)	0.26124	18	[9,−19]
(C6, R5)	0.45302	15	[17,−6]
